# Metal-Dependent Cell Death in Renal Fibrosis: Now and in the Future

**DOI:** 10.3390/ijms252413279

**Published:** 2024-12-11

**Authors:** Te Li, Chen Yu

**Affiliations:** Department of Nephrology, Tongji Hospital, School of Medicine, Tongji University, Shanghai 200065, China

**Keywords:** metal ions, cell death, cuproptosis, ferroptosis, zinc, renal fibrosis

## Abstract

Renal fibrosis is a common final pathway underlying nearly almost all progressive kidney diseases. Metal ions are essential trace elements in organisms and are involved in important physiological activities. However, aberrations in intracellular metal ion metabolism may disrupt homeostasis, causing cell death and increasing susceptibility to various diseases. Accumulating evidence suggests a complex association between metal-dependent cell death and renal fibrosis. In this article, we provide a comprehensive overview of the specific molecular mechanisms of metal-dependent cell death and their crosstalk, up-to-date evidence supporting their role in renal fibrosis, therapeutic targeting strategies, and research needs, aiming to offer a rationale for future clinical treatment of renal fibrosis.

## 1. Introduction

Renal fibrosis, a common outcome of nearly all progressive kidney diseases, is characterized by morphological features such as glomerulosclerosis, tubular atrophy, interstitial chronic inflammation, fibrogenesis, and vascular rarefaction. Fibrosis arises from the dysregulation of wound healing processes, resulting in the excessive accumulation of extracellular matrix (ECM) proteins, including fibronectin and collagens. Upon kidney injury, local fibroblasts and pericytes are activated, enhancing their contractility, secreting inflammatory mediators, and synthesizing ECM components, thereby initiating the wound healing response. However, if damage to the kidneys is repetitive or severe, the persistent accumulation of ECM proteins can impair tissue repair capacity, leading to renal dysfunction and potentially resulting in organ failure [1,2,3]. Despite significant advances in understanding the underlying mechanisms of renal fibrosis, effective therapeutic strategies remain elusive.

Recent studies have underscored the potential role of metal ions, particularly iron and copper, in the pathogenesis of kidney diseases, garnering increasing attention [4]. Metal ions are essential trace elements, participate in key physiological activities, and help maintain homeostasis [5]. For instance, various enzymes require metal ions as cofactors for their proper function [6]. However, disruptions in intracellular ion balance can lead to excessive cellular damage and death, ultimately contributing to a spectrum of various diseases and health issues [7]. Ferroptosis, a regulated form of cell death, is characterized by iron-dependent lipid peroxidation accumulation [8]. Cuproptosis is a process in which excess intracellular copper induces the degradation of iron–sulfur (Fe-S) cluster proteins and proteotoxic responses due to the aggregation of mitochondria-associated proteins, ultimately leading to cell death [9]. Other metal ions, such as zinc, can trigger oxidative stress and reactive oxygen species (ROS), thereby inducing cell damage and death [10,11]. This form of programmed cell death (PCD), initiated by metal ions, is termed metal-dependent cell death.

Growing evidence underscores an intricate relationship between metal-dependent cell death and renal fibrosis [12,13,14,15]. For example, Zhang et al. have indicated that lipoxstatin-1, a ferroptosis inhibitor, attenuates unilateral ureteral obstruction (UUO)-induced renal fibrosis [16]. Similarly, ferrostatin-1 has been demonstrated to mitigate oxalate-induced renal fibrosis by inhibiting ferroptosis [17]. Although the relationship between renal fibrosis and metal-dependent cell death has been preliminarily recognized, further research is imperative to understand the specific contribution of metal-dependent regulated cell death to renal fibrosis. This review provides a comprehensive overview of the specific molecular mechanisms underlying metal-dependent cell death and their crosstalk, presents evidence supporting their role in renal fibrosis, outlines targeted therapeutic strategies, and identifies current research needs, aiming to offer a rationale for future clinical treatment of renal fibrosis.

The search strategy for this review, “metal-dependent cell death, ferroptosis, cuproptosis, cell death, iron, copper, zinc, renal fibrosis” was used in PubMed, Medline, and Web of Science as keywords. The search time was from the establishment of the database to 30 September 2024. The literature inclusion criterion was basic research and literature research based on metal-dependent cell death in renal fibrosis. The literature exclusion criteria were repeated publication, lack of detailed data records, too old, and poor-quality studies.

## 2. Metal Ions Are Deeply Involved in Cell Death

Metal ions are deeply involved in maintaining cellular homeostasis, each with unique reactivity and mechanism of action. However, disturbances in intracellular metal ion metabolism can impair homeostasis, contributing to cell death and a spectrum of diseases, including neurodegenerative disorders [18,19], cancer [20], cardiovascular disease [21], and renal fibrosis [22].

### 2.1. Iron Metabolism and Ferroptosis

Iron, the most abundant micronutrient in the human body, is essential for driving normal biological processes [23]. Iron metabolism is stringently regulated at both cellular and systemic levels to maintain iron concentrations within the optimal physiologic range. This balance is achieved through the precise expression and activity of iron carriers, transporters, and proteins involved in regulation and storage [24]. Iron is primarily obtained from diet and is absorbed predominantly in the duodenum and small intestine. Macrophages in the spleen, liver, and bone marrow achieve iron recycling via phagocytosis of senescent red blood cells [25]. Dietary ferric iron (Fe^3+^) is first reduced to ferrous iron (Fe^2+^) by ferrireductases (such as duodenal cytochrome B) and possibly other reductases, and transported into the cells via divalent metal-ion transporter 1 (DMT1) [26,27]. Intracellular Fe^2+^ is exported via ferroportin located on the basolateral membrane and oxidized to Fe^3+^ with the help of hepcidin and ceruloplasmin, allowing it to bind to transferrin for circulation [4]. The majority of cellular iron is stored in ferritin, while a portion is in labile iron pools (LIPs). Mitoferrin, siderofexin, and DMT1 can transport Fe^2+^ stored from the LIPs to the mitochondria [28]. Excess intracellular iron from the LIP can be stored in lysosomes as ferritin. Iron is typically present as Fe^2+^ within lysosomes due to their acidic and reducing conditions. When intracellular iron ions are reduced, the autophagic degradation of ferritin in lysosomes can release divalent iron to replenish LIP [29]. A relatively stable LIP is meticulously maintained by a sophisticated regulatory mechanism of intracellular iron absorption, storage, and transport [15,30]. Intracellular iron homeostasis is regulated by iron response elements and iron regulatory proteins (IRPs). IRP1 is regulated by the iron-dependent synthesis of Fe-S clusters. IRP2 undergoes ubiquitin-mediated degradation by proteins such as partner F-box and leucine-rich repeat protein 5, whose mode of action and stability are influenced by Fe-S clusters and oxygen levels [31,32]. Systemic iron homeostasis is managed by hepcidin [33]. The bioactive hormone operates by binding directly to ferroportin, inducing its endocytosis, ubiquitination, and iron exporter degradation in lysosomes, thus decreasing available circulating iron [33,34]. In cases of iron overload, the excess circulating iron can exceed the binding capacity of transferrin, leading to the formation of non-transferrin-bound iron [23]. Free Fe^2+^ can readily react with H_2_O_2_ in the Fenton reaction, catalyzing the generation of ROS and causing cellular oxidative damage and ferroptosis [35].

**Figure 1:**
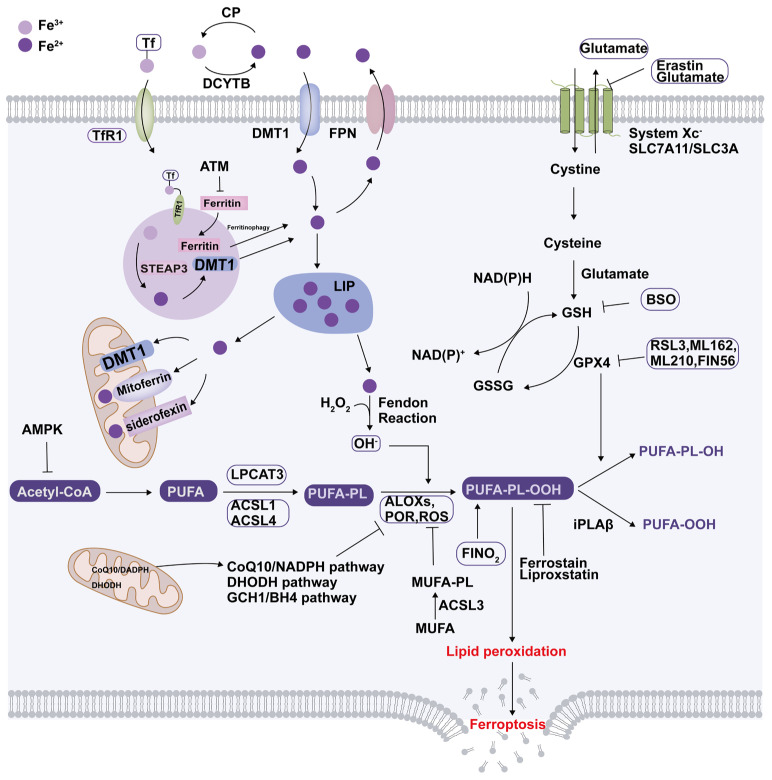
Iron metabolism and ferroptosis. Extracellular irons predominantly exist in the form of Fe^3+^. Fe^3+^ is first reduced to Fe^2+^ by ferrireductases, transporting it into the cells via DMT1. Intracellular Fe^2+^ is exported via ferroportin located on the basolateral membrane and oxidized to Fe^3+^ with the help of ceruloplasmin, allowing it to bind to transferrin for circulation. The majority of cellular iron is stored in ferritin, while a portion is in LIPs. Mitoferrin, siderofexin, and DMT1 can transport Fe^2+^ stored from the LIPs to the mitochondria. Excess intracellular iron from the LIP can be stored in lysosomes as ferritin. Iron is typically present as Fe^2+^ within lysosomes due to their acidic and reducing conditions. When intracellular iron ions are reduced, the autophagic degradation of ferritin in lysosomes can release divalent iron to replenish LIP. Ferroptosis is characterized by the depletion of intracellular glutathione and decreased activity of GPX4, which leads to the accumulation of unmetabolized lipid peroxides and increased ROS production. Membrane damage is also a result of lipid peroxidation. Moreover, the FSP1/CoQ10/NAD(P)H axis, the GCH1/BH_4_ axis, and the DHODH pathways suppress ferroptosis in parallel with the GPX4-dependent pathway. ALOXs, lipoxygenases; AMPK, adenosine-monophosphate-activated protein kinase; ACSL, acyl-CoA synthetase long-chain family member; ATM, ATM serine/threonine kinase; BSO, buthionine sulfoximine; CP, ceruloplasmin; CoA, coenzyme A; CoQ10, coenzyme CQ10; DCYTB, duodenal cytochrome B; DHODH, dihydroorotate dehydrogenase (quinone); DMT1, divalent metal-ion transporter 1; FPN, ferroportin; FSP1, ferroptosis suppressor protein 1; GCH1, GTP cyclohy drolase 1; GCLC, glutamate-cysteine ligase catalytic subunit; Glu, glutamate; GPX4, glutathione peroxidase 4; GSH, glutathione; iPLA2b, phospholipase A2 group VI; LIP, labile iron pools; LPCAT3, lysophosphatidylcholine acyltransferase 3; MUFA, monounsaturated fatty acid; NADPH, reduced nicotinamide adenine dinucleotide phosphate; PL, phospholipid; POR, Cytochrome p450 oxidoreductase; PUFA, polyunsaturated fatty acid; PUFA-PL-OOH, phospholipid with peroxidized polyunsaturated fatty acyl tail; ROS, reactive oxygen species; System xc-, sodium-independent, anionic amino acid transport system; ATSTEAP, six-transmembrane epithelial antigen of the prostat; Tf, transferrin; TfR1, transferrin receptor protein 1.

Ferroptosis, first described by Brent Stockwell’s lab in 2012, is a special cell death mode typified by the accumulation of iron and lipid peroxidation [8]. Its susceptibility is dependent on lipid peroxidation, iron metabolism, and the cellular antioxidant defense system [36]. The accumulation of lipid peroxides produced by the oxidation of polyunsaturated fatty acids (PUFAs) is a hallmark of ferroptosis [37,38]. However, monounsaturated fatty acids can suppress the process, suggesting that not all oxidized lipids promote ferroptosis [36,39]. They suppress lipid ROS accumulation and inhibit ferroptosis by free fatty acid-activating enzymes such as acyl-CoA synthetase long-chain family member 3 (ACSL3) [40]. The biosynthesis of PUFAs is governed by a multitude of enzymes and metabolic pathways. PUFA-containing phospholipids, which serve as the principal substrates for lipid peroxidation, are positively regulated by several enzymes such as ACSL1, ACSL4, lysophosphatidylcholine acyltransferase 3, P450 oxidoreductase, and lipoxygenases family (like ALOXs) [41,42,43,44,45]. Excess intracellular iron amplifies the process of lipid peroxidation via the Fenton action, leading to the generation of ROS and hydroxyl radicals. These reactive species initiate attacks on cell membranes and cause damage, which in turn triggers cell death [46]. Both ferritin degradation in lysosomes and transferrin receptor 1 expression regulate ferroptosis by modulating ROS-induced autophagy and iron abundance [47]. Inhibition of ATM serine/threonine kinase can rescue ferroptosis by upregulating the expression of iron regulatory proteins critical for iron storage, such as ferritin [48]. In contrast, glutathione peroxidase 4 (GPX4) is currently known to be a key repressor of ferroptosis, functioning in concert with glutathione (GSH) to maintain cellular redox balance [49]. The maintenance of intracellular GSH concentration homeostasis helps to neutralize free radicals and ROS, protecting cells from oxidative stress and lipid peroxidation [50]. The activation of the cystine–glutamate antiporter system, comprising solute carrier family 7a member 11 (SLC7A11) and SLC3A2, imports cystine and glutamate–cysteine ligase to promote GSH biosynthesis, exerting a pivotal influence on ferroptosis regulation [51]. In addition to inhibiting the activity of GPX4 (such as FINO_2_, RSL3, ML162, and ML210), the degradation of GPX4 (such as FIN56) can also promote ferroptosis [52,53]. While GPX4 is a pivotal suppressor of ferroptosis, GPX4-independent mechanisms also exist. IPLA2β inhibits p53-induced repression of ferroptosis in a GPX4-independent manner [54]. The ferroptosis suppressor protein 1 (FSP1)/coenzyme Q10 (CoQ10)/nicotinamide adenine dinucleotide phosphate (NAD(P)H) axis, the GTP cyclohydrolase 1 (GCH1)/tetrahydrobiopterin (BH_4_) axis, and the dihydroorotate dehydrogenase (DHODH) pathway suppress ferroptosis in parallel with the GPX4-dependent pathway [55,56,57,58,59]. Moreover, ferroptosis is indivisibly connected to the dysregulation of redox homeostasis. With the exception of GSH, nuclear factor erythroid 2-related factor 2 (Nrf2) is also a master transcription factor in the antioxidant system. Nrf2 activates the expression of proteins and enzymes that protect against lipid peroxidation, including the SLC48A1 and heme-oxygenase-1 (HO-1), which are integral to the defense against ferroptosis [60,61] (Figure 1).

### 2.2. Copper Metabolism and Cuproptosis

Copper is an essential trace element for numerous organisms, acting as a cofactor or structural component in a variety of enzymes involved in cellular processes [62]. It is primarily derived from food, with the majority of copper being absorbed in the duodenum and small intestine [63]. Once absorbed, copper is released into the bloodstream, where approximately 90% is bound to plasma ceruloplasmin. The remaining portion of copper is bound to soluble partner proteins, such as histidine-rich proteins and albumin [64]. The portal vein transports absorbed copper to the liver, which is the primary site for copper capture, distribution, and excretion, thus playing a central role in regulating the body’s copper balance [65]. Copper can be stored in hepatocytes, excreted into bile, or released back into the bloodstream for further distribution to other tissues. A minor fraction of copper is excreted through sweat, urine, and menstrual fluid [66].

Extracellular copper ion exists as Cu^2+^, which enters the cell via DMT1. Cu^2+^ is converted to Cu^+^ via the duodenal cytochrome b (DCYTB) and six-transmembrane epithelial antigen of the prostate (STEAP) on the intestinal cell membrane since Cu^2+^ cannot be directly absorbed by epithelial cells and hepatocytes. Copper affinity transporter 1 (CTR1) transports Cu+ into cells [62,67]. CTR1 is localized to both the plasma membrane and intracellular vesicles, with cellular copper levels modulating its trafficking between these locations [68]. Within the cytoplasm, Cu^+^ can be chelated by metallothionein or transported to various organelles by copper chaperone proteins. For example, Cu^+^ is transferred to the mitochondria via cytochrome C oxidase 17 (COX17). COX17 is responsible for binding copper within the intermembrane space of mitochondria and facilitating its transfer to either synthesis cytochrome c oxidase 1 (SCO1) or COX11. Subsequently, COX11 transfers copper to cytochrome c oxidase (CCO), where it plays a role in oxidative respiratory and redox processes [69]. Additionally, copper can be transported to superoxide dismutase 1 (SOD1) via the copper chaperone for superoxide dismutase (CCS), thereby regulating its localization in the cytoplasm and mitochondria [70]. Additionally, copper interacts with antioxidant 1 copper chaperone (ATOX1), which facilitates its transport to the Golgi network ATP-7A/7B enzyme (ATP 7A/7B), while some copper molecules bind transcription factors in the nucleus to modulate gene expression [71]. ATPases of the 7A and 7B subtypes, also known as P-type Cu-ATPases, utilize the hydrolysis of ATP to facilitate the export of excess copper and regulate copper homeostasis [72]. Copper metabolism must be rigorously regulated to ensure the maintenance of cellular homeostasis and the prevention of cellular damage, death, and disease.

**Figure 2:**
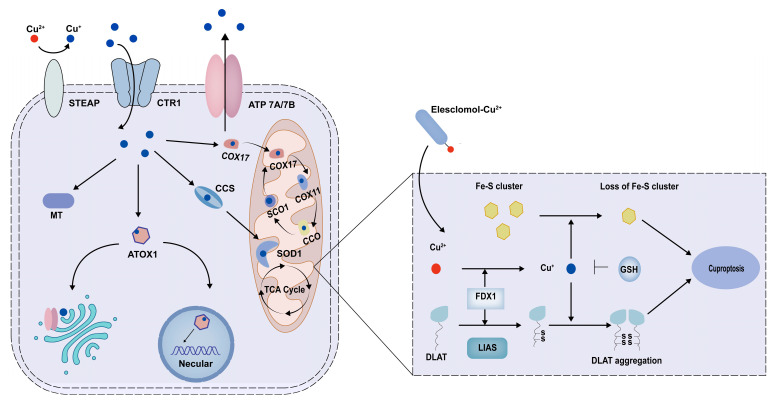
Copper metabolism and cuproptosis. Extracellular copper ions predominantly exist in the form of Cu^2+^. Cu^2+^ is reduced to Cu^+^ via the action of STEAP on the intestinal cell membrane. The cellular uptake of Cu+ is facilitated by CTR1, whereas its export is mediated by ATP7A and ATP7B. Intracellularly, Cu+ is directed to various subcellular organelles for bioavailability by copper-binding proteins, including COX17, CCS, and ATOX1. Additionally, the binding of MT to copper can mitigate the cytotoxicity associated with copper excess. Elesclomol binds Cu^2+^ in the extracellular environment and transports it to mitochondria. Cu+ directly binds to DLAT, promoting protein acylation and inducing the degradation of Fe-S cluster proteins, which ultimately induces proteotoxic stress and cell death. GSH serves as a thiol-containing copper chelator that blocks cuproptosis. STEAP, six-transmembrane epithelial antigen of the prostate; CTR1, copper affinity transporter 1; ATP7A/7B, ATPases of the 7A and 7B; COX17, cytochrome C oxidase 17; SCO1, synthesis cytochrome c oxidase 1; CCO, cytochrome c oxidase; SOD1, superoxide dismutase 1; CCS, copper chaperone for superoxide; ATOX1, antioxidant 1 copper chaperone; MT, metal-ion transporter; TCA cycle, mitochondrial tricarboxylic acid cycle; FDX1, ferredoxin 1; DLAT, dihydrolipoamide S-acetyltransferase; LIAS, lipoic acid synthetase; GSH, glutathione. Fe-S, iron-sulfur.

Cuproptosis, initially proposed by Peter Tsvetkov et al. in 2022, is a special form of copper-induced cell death, distinct from other known regulated cell deaths such as apoptosis, pyroptosis, and necroptosis [9]. Through gene screening and knockout detection, they identified that mitochondrial matrix reductases ferredoxin 1 (FDX1) and lipoic acid synthetase (LIAS) are pivotal regulators of cuproptosis. FDX1, an upstream regulator of protein lipoylation, contributes to the accumulation of lipoylated dihydrolipoamide S-acetyltransferase (DLAT). Additionally, FDX1 functions as a catalyst for the reduction of intracellular Cu^2+^ to Cu^+^, which is then released into the mitochondria. LIAS, a component of the lipoic acid pathway, plays a key role in regulating the mitochondrial tricarboxylic acid (TCA) cycle. DLAT is a critical enzyme in the TCA cycle and is essential for the lipoacylation of proteins. In summary, intracellular Cu+ directly binds to DLAT, promoting protein acylation and inducing the degradation of Fe-S cluster proteins, which ultimately induces proteotoxic stress and cell death (Figure 2).

### 2.3. Other Metal Ions

Zinc is a significant micronutrient that is required for a variety of physiological activities. Dietary zinc is primarily absorbed in the intestine, with intestinal epithelial cells taking up the zinc and transporting it to peripheral tissues such as skeletal muscle (60%), bone (30%), skin (5%), and other tissues (5%) [73]. Bioinformatic analyses indicate that approximately 10% of human proteins are capable of zinc binding, underscoring its essential role in diverse physiological processes [74]. The maintenance of zinc homeostasis relies on a complex, coordinated system of proteins involved in zinc uptake, excretion, storage, and transport [75]. Metallothionein has been demonstrated to bind zinc, thereby protecting cells from oxidative stress [76]. The zrt- and Irt-like proteins (ZIPs) and zinc transporters (ZnTs) are two families of zinc transporters responsible for regulating intracellular zinc homeostasis [77]. ZnT primarily facilitates zinc influx into cells, whereas ZIPs mediate the efflux of excess zinc ions [78]. Both zinc deficiency and excessive absorption can disrupt zinc homeostasis, triggering various cellular responses and potentially leading to cell death.

Zinc has been shown to inhibit apoptosis, and its deficiency is associated with the induction of PCD across a range of cell lines [79]. Zinc deficiency reduces growth factor signaling pathways and enhances p53 pathway activation, mediated by hypo-phosphorylation of protein kinase B (AKT) and extracellular regulated protein kinases (ERKs). Additionally, low zinc concentrations increase the generation of ROS and reactive nitrogen species, as well as caspase activation. Furthermore, zinc deficiency impairs the transactivation potential of endogenous survival pathways such as NF-kB. These effects collectively contribute to the induction of apoptosis in zinc-deficient [80]. However, excessive zinc overload can also induce cell death, highlighting the importance of zinc homeostasis for cellular health. Excessive zinc activates the PINK1/Parkin signaling pathway and elevates lactate dehydrogenase and ROS levels, inducing cell death, mitochondrial autophagy, and mitochondrial dysfunction [81]. Lysosomes, in conjunction with mitochondria, serve as important intracellular zinc reservoirs. The release of zinc from the lysosomes can trigger rapid mitochondria-mediated non-apoptotic cell death [82]. In neuronal and astrocyte cells, excess zinc rapidly activates neuronal nitric oxide synthase, protein kinase C, and ERK1/2, increasing oxidative stress and over-activating poly (ADP-ribose) polymerase, which can deplete NAD+/ATP levels. Zinc can also deplete ATP levels by inhibiting glycolysis and glyceraldehyde-3-phosphate dehydrogenase, potentially causing the occurrence of necrosis [83]. The precise mechanisms of underlying cell death induced by zinc deficiency or overload remain unclear and warrant further in-depth investigation to elucidate their impact on cellular homeostasis and survival.

It has become increasingly clear that perturbations in metal ion homeostasis can precipitate a variety of cell death pathways. Regrettably, other highly specific metal-dependent cell death pathways analogous to ferroptosis and cuproptosis remain to be identified.

## 3. Crosstalk Among Different Metal-Dependent Cell Deaths

### 3.1. Mitochondria Is a Key Organelle

As the primary organelle responsible for maintaining the normal physiological activities of cells, mitochondria are of importance in the regulation of energy metabolism and PCD. Metal-dependent cell death as a subset of PCD is intricately connected to mitochondrial metabolism.

Mitochondria are indispensable in the execution of ferroptosis. First, mitochondrial morphology is notably altered in cells undergoing ferroptosis, including reduced membrane density and volume, diminished or vanished cristae, and outer membrane ruptures. In addition to morphological alterations, ferroptosis impairs mitochondrial activity, which in turn results in a reduction in ATP generation, DNA damage, and a decrease in heme synthesis [84]. Secondly, mitochondrial DHODH and CoQ10 suppress ferroptosis via the GPX4-independent pathway [39,85]. Thirdly, the inhibition of the mitochondrial electron transport chain (ETC) has been demonstrated to mitigate ferroptosis induced by cysteine starvation, a process that is analogous to the depletion of mitochondria [86]. Glutaminolysis, which replenishes the mitochondrial TCA cycle, is implicated in supporting deprivation-induced ferroptosis through the promotion of lipid peroxidation [87]. This highlights the crucial function of both the TCA cycle and ETC in the fundamental processes leading to cysteine deprivation-induced ferroptosis. Last but not least, mitochondria are the primary sources of ROS, which play a critical role in the process of ferroptosis. Excessive mitophagy can lead to the release of iron, undergoing a Fenton reaction and consequently producing substantial ROS. These excess iron ions and ROS initiate a chain reaction with PUFAs, resulting in uncontrollable lipid peroxidation and the eventual onset of ferroptosis [88].

Cuproptosis represents a further type of metal-dependent cell death occurring within the mitochondria. During cuproptosis, significant mitochondrial morphology alterations are observed, such as shrinkage and membrane rupture. Cuproptosis is mediated by lipoylation of proteins, particularly enzymes involved in the mitochondrial TCA cycle [89]. The susceptibility of cells with high reliance on mitochondrial respiration to cuproptosis is greater [90]. Notably, the alterations in mitochondrial respiration and protein lipoylation during cuproptosis can be suppressed by the respiratory chain complex inhibitors I and III (such as antimycin and rotenone A), as well as the mitochondrial pyruvate transporter inhibitor (such as UK5099) [91]. The occurrence of cuproptosis disrupts normal mitochondrial metabolic processes. While mitochondrial components are theoretically eliminated via lysosomal degradation, the capacity of mitophagy to directly prevent cuproptosis is yet to be established. Elevated intracellular copper levels can also lead to the production of ROS through the Fenton reaction. Nevertheless, it remains unclear whether the remaining mitochondria can sustain normal energy metabolism after cuproptosis, necessitating further investigation into the long-term effects of cuproptosis on cellular energetics.

As a key cofactor in SOD1, zinc is involved in the synthesis of enzymes for mitochondrial energy metabolism, such as COX [92]. Zinc deficiency impairs the antioxidant capacity of SOD1 and activates the p53 signaling pathway, which in turn results in increased ROS production and subsequent apoptosis [10]. Zinc supplementation can reverse this process, as indicated by studies showing its ability to modulate the ROS/NF-κB pathway and reduce oxidative stress [93]. However, excessive zinc can induce oxidative stress and ROS generation, creating a positive feedback loop whereby zinc-binding proteins are oxidated, resulting in further zinc release and accelerating cell death. Additionally, elevated zinc levels can induce a form of non-apoptotic cell death characterized by mitochondrial swelling and the inhibition of ATP synthesis [94].

In conclusion, mitochondria play a key role in metal-dependent cell death, affecting the cell death process through their central function in cellular metabolism. Conversely, various cell death pathways can also result in mitochondrial structural damage and dysfunction. The mitochondrial TCA cycle appears to be a potential intersection between ferroptosis and cuproptosis, yet the exact nature of this link remains to be fully elucidated. Further studies are required to clarify the mechanisms underlying metal-dependent cell death and its intricate association with mitochondrial function. Achieving a comprehensive understanding of the mitochondrial process is crucial for deciphering the mechanisms of metal-dependent cell death, its regulatory process, and its implications for various diseases.

### 3.2. GSH Is a Common Hub

GSH is considered the paramount antioxidant within the intracellular antioxidant system. It neutralizes oxidative substances and prevents oxidative stress, maintaining intracellular redox homeostasis. GSH depletion disrupts this balance, ultimately triggering cell dysfunction and death [95]. GSH has been demonstrated to inhibit lipid peroxidation, thereby preventing the initiation of ferroptosis. In cuproptosis, GSH performs the function of copper chaperone, binding to copper to prevent the aggregation of Fe-S proteins and subsequent cellular damage. Notably, GSH has been demonstrated to suppress both ferroptosis and cuproptosis, indicating its role as a mediator in the crosstalk among these processes. The synthesis and regulation of GSH may represent a common mechanism underlying both types of metal-dependent cell death.

P53, the most frequently mutated tumor suppressor gene, is involved in metal-dependent cell death. P53-mediated transcriptional upregulation of cyclin-dependent kinase inhibitor 1A suppresses ferroptosis in a GSH-dependent manner. Zinc deficiency activates the p53 pathway, causing ROS accumulation and apoptosis. Nevertheless, the function of P53-regulated GSH in cuproptosis is still uninvestigated, and the hypothesis that p53 can regulate cuproptosis is awaiting further experimental validation.

Buthionine sulfoximine (BSO) is an inhibitor of GSH synthesis that has been shown to induce cuproptosis by depleting the endogenous copper chelator GSH [9]. Similarly, BSO inhibits GSH-dependent peroxidase activity, leading to increased lipid peroxidation and the induction of ferroptosis. Consequently, BSO is capable of targeting both ferroptosis and cuproptosis, which may render it a promising therapeutic agent. Nevertheless, further study is essential for fully clarifying the mechanisms by which BSO engages with these pathways, thereby enhancing the clinical applicability of this approach.

## 4. Metal-Dependent Cell Death in Renal Fibrosis

Imbalance in intracellular metal ions cause cell death and tissue damage and contributes to the development of renal fibrosis (Table 1). Since ferroptosis was proposed in 2012, it has been extensively studied, including its association with renal fibrosis. In vivo experiments frequently utilize UUO models to induce renal fibrosis with the primary tubular injury due to obstructed urine flow. Liproxstatin-1 is a well-recognized ferroptosis inhibitor that offers protection against agents that induce ferroptosis, including BSO, erastin, and (1S,3R)-RSL3. Liproxstatin-1 reduces surrounding fibroblast activation by preventing the paracrine secretion of profibrotic factors in HK2 cells, thereby attenuating UUO-induced renal fibrosis by preventing ferroptosis-mediated renal tubular epithelial cell death [16]. In addition, the Astragalus mongholicus Bunge and Panax notoginseng formula, a self-developed refined traditional Chinese medicine (TCM) formulation, has demonstrated efficacy in alleviating inflammation associated with various acute and chronic kidney diseases (CKDs). Recent studies have shed light on its potential mechanisms in improving renal fibrosis in UUO mice by inhibiting the downregulation of ferroptosis signaling [96]. Similarly, kaempferitrin, a flavonoid glycoside derived from Bauhinia leaf, possesses established anti-inflammatory properties, and recent research has identified it as a potential nephroprotective agent capable of inhibiting NADPH oxidase 4 (NOX4)-mediated ferroptosis in tubular cells [97]. Nobiletin, a distinct dietary flavonoid found in citrus fruit peels, has demonstrated anti-ferroptosis and antifibrotic effects, effectively attenuating the development of renal fibrosis in the UUO model [98]. Furthermore, tectorigenin, a principal component isolated from Belamcanda chinensis, exhibits diverse pharmacological functions, notably its nephroprotective effects. It has been shown to mitigate renal fibrosis induced by UUO through the inhibition of Smad3-mediated ferroptosis [99]. As a potential antioxidant. Baicalein exerts anti-fibrosis effects by decreasing the ferroptosis response [100]. In renal fibrosis, the increased secretion of interleukin-6 (IL-6) exacerbates renal inflammation. Moreover, IL-6 is implicated in promoting the fibrotic response by promoting the production of collagen. Tocilizumab is an emerging therapy for conditions mediated by IL-6, and tocilizumab mimotope has demonstrated the ability to alleviate renal fibrosis by inhibiting ferroptosis in the UUO model, presenting a potential alternative therapy for renal fibrosis [101]. In studies, folic acid (FA) is also commonly used to induce renal fibrosis. FA-induced kidney injury is characterized by tubule damage, resulting in the disruption of the antioxidant system, leading to subsequent interstitial fibrosis. Formononetin, a bioflavonoid derived from herbs such as Astragalus radix, *Trifolium pratense*, and other traditional Chinese medicines, is known for its antioxidant properties and its ability to reduce ROS. Formononetin can suppress the Smad3/activating transcription factor 3/SLC7A11 signaling pathway, thereby ameliorating renal fibrosis induced by UUO and FA [102]. FG-4592 is an inhibitor of hypoxia-inducible factor prolyl hydroxylase. The protective role of FG-4592 is primarily attributed to its ability to reduce the occurrence of ferroptosis in the early stages of FA-induced renal injury by activation of the AKT/Glycogen synthase kinase 3β-mediated Nrf2 pathway, which slows the development of fibrosis [103].

Diabetic nephropathy (DN) is a prevalent renal disease in individuals with diabetes, representing a serious microvascular complication of the disease. Renal fibrosis is the primary pathological manifestation [134]. Various studies have indicated that the occurrence and progression of DN are deeply associated with ferroptosis. Inhibition of ferroptosis through different pathways can alleviate the progression of DN, providing a theoretical basis for DN treatment. For example, fibrotic area and collagen I levels increased in DN mice compared to normal mice, indicating increased renal fibrosis. However, treatment with ferrostatin-1, a ferroptosis inhibitor, has been shown to mitigate these effects [104]. Moreover, TCM has been widely used in the prevention and treatment of DKD, offering notable efficacy and minimal adverse reactions. Studies focused on natural active substances or extracts with anti-inflammatory and anti-oxidation properties. For example, hederagenin, a naturally occurring pentacyclic triterpenoid found in plants like ivy and Pulsatilla, has been reported to exhibit diverse biological activities. Specifically, it ameliorates renal fibrosis in DN by suppressing Smad3/NOX4/SLC7A11-mediated ferroptosis in tubular cells [105]. Ginkgolide B is one of the natural bioactive components of Ginkgo biloba extract. Research has demonstrated that Ginkgolide B mitigates free radical damage, curbs inflammatory responses, and inhibits ferroptosis, thereby exerting a protective effect on the DN [106]. Similarly, Rhein is a naturally occurring anthraquinone derivative, which is predominantly sourced from the rhizomes of plants in the genus Rheum. It inhibits ferroptosis and attenuates DN via controlling the ras-related C3 botulinum toxin substrate 1/NOX1/β-catenin axis [107]. Shenkang Pills are a TCM formula that has demonstrated the potential to mitigate renal fibrosis in DN by suppressing ferroptosis via the hypoxia-inducible factor-1α/HO-1 pathway [108]. Of course, the treatment of DN is not limited to traditional Chinese medicine. Atorvastatin is a commonly prescribed medication in clinical practice for the reduction of blood lipids and cholesterol. Currently, there is evidence that it has a beneficial effect on DN by suppressing oxidative stress and ferroptosis [109]. The advent of next-generation sequencing (NGS) technology has facilitated a plethora of significant discoveries in genomics research. Studies have shown a range of differentially expressed tRNA-derived fragments (a class of small non-coding RNAs generated by specific nucleases) within the context of DN. tRF3-IleAAT is a tRNA-derived fragment that reduces the synthesis of ECM in DN mice by targeting zinc finger protein 281 and inhibiting ferroptosis, suggesting its potential as an attractive therapeutic target for DKD [110]. Epithelial–mesenchymal transition (EMT) in renal tubular epithelial cells can cause tubulointerstitial fibrosis. ER stress, an important stimulant during EMT progression, induces ferroptosis-related EMT development through the X-box binding protein 1/HMG-CoA reductase degradation protein 1/Nrf2 pathway, thereby causing the progression of renal fibrosis in DN [111].

It is estimated that 10–14% of the population worldwide is impacted by chronic kidney disease (CKD), with renal fibrosis representing a hallmark manifestation in various progressive forms of CKD. Despite the absence of antifibrotic therapies for CKD, increasing evidence suggests a robust relationship between CKD and ferroptosis [2]. For instance, ferroptosis plays a role in the progression of renal fibrosis induced by a 5/6 nephrectomy rat model [13]. As CKD disease progresses, the toxin levels in patients increase further. The uremic toxin indoxyl sulfate can trigger intracellular iron accumulation and ROS generation, resulting in ferroptosis and accelerating the time to enter end-stage renal disease [114]. In addition, targeted gene therapy is gradually moving towards the stage of CKD treatment. Homeobox D10, identified as a tumor suppressor, plays a pivotal role in nephrogenesis and renal development. Recent research has found that homeobox D10 alleviates CKD-related fibrosis by inhibiting the transcription of NOX4, the activation of the ferroptosis pathway, and the expression of the profibrotic gene [112]. Additionally, TCM therapy is well recognized in the treatment of CKD. Vitexin, a naturally occurring flavonoid, possesses diverse pharmacological properties, including antioxidant, anti-inflammatory, and protective effects against various diseases, including the amelioration of renal fibrosis in CKD. It has been shown to attenuate CKD-induced fibrosis by activating the Nrf2/HO-1 axis, which in turn increases expression levels of GPX4 and further suppresses lipid peroxidation and ferroptosis [113]. In addition, Fisetin, as a natural product, is widespread in nuts, fruits, as well as vegetables. It exhibits protective effects against CKD-induced renal fibrosis by inhibiting ACSL4-mediated tubular ferroptosis [115].

Hypertensive nephropathy is the main cause of end-stage renal disease. Persistent hypertension results in injury to tubular cells, thereby contributing to tubulointerstitial fibrosis [135]. Sirtuin7 belongs to class III histone deacetylase; remarkably, it can antagonize TGF-β signaling and suppress developmental EMT. Thus, Srtuin7 was recently demonstrated to be able to alleviate renal ferritin deposition and lipid peroxidation under conditions of hypertension, thereby reducing renal fibrosis [116]. The expression of stimulators of interferon genes is elevated in patients with hypertensive nephropathy, maybe because the overexpression of stimulators of interferon genes can transduce damage signals through ACSL4-dependent ferroptosis to promote fibrosis [117]. Elevated oxalic acid and uric acid can easily lead to kidney stone formation, both closely linked to renal fibrosis [136,137]. Ferrostatin-1 alleviates hyperuricemic nephropathy and oxalate-induced renal fibrosis by suppressing ferroptosis [17,118].

Renal interstitial fibrosis is a key pathological characteristic of chronic renal allograft dysfunction. Ferroptosis can contribute to tubular injury, which causes the release of profibrotic factors and facilitates renal interstitial fibrosis progression during chronic renal allograft dysfunction [119]. Moreover, melatonin, a potent antioxidant, and zileuton, known for its free radical scavenging capacity, have emerged as promising therapeutic drugs for kidney injury induced by ferroptosis. Research has demonstrated that the combination of melatonin and zileuton alleviates ferroptosis-related renal fibrosis by enhancing the activity of the AKT/mTOR/Nrf2 pathway [120]. Renal aging is marked by the accumulation of senescent cells and chronic inflammation, leading to the progression of renal interstitial fibrosis and a decline in renal function. A study has shown that iron dyshomeostasis in aging macrophages is crucial in both ferroptosis and renal fibrosis [121]. Low-intensity pulsed ultrasound, a non-invasive therapeutic modality delivered in pulse wave mode, has recently received increased attention for its potential role in modulating the Nrf2/Keap1/HO-1 pathway, thereby effectively suppressing renal fibrosis [122,138]. Ischemia-reperfusion (IR) injury, a major cause of acute kidney injury, frequently results in subsequent renal fibrosis. Puerarin can alleviate ferroptosis induced by IR injury through the toll-like receptor 4/NOX4 pathway [123]. Obesity, a prevalent global public health concern, is strongly associated with a range of diseases, including renal fibrosis. Evidence suggests that ferrostatin-1 treatment attenuates high-fat diet-induced renal fibrosis [124].

Not only is ferroptosis strongly associated with renal fibrosis, but the imbalance of other metal ions also contributes to the condition. Intracellular copper overload exacerbates cytotoxicity and accelerates renal fibrosis progression. For example, elevated intracellular copper induced by CTR1 activates lysyl oxidase, enhancing collagen and elastin crosslinking, which promotes renal fibrosis [22]. Similarly, the mitochondria serve as both major sites of intracellular copper utilization and copper reservoirs. Excessive mitochondrial copper impairs energy metabolism, which in turn generates mitochondrial dysfunction, cellular senescence, and renal fibrosis [125]. COX17, the copper chaperone protein, attenuates renal fibrosis through the maintenance of mitochondrial copper homeostasis and restoration of respiratory chain complex IV activity [126]. Zinc, as an essential dietary element, plays multiple roles in preventing renal fibrosis. Severe renal cell necrosis due to zinc deficiency can aggravate fibrosis by promoting kidney inflammation [127]. Zinc deficiency also contributes to podocyte damage, which subsequently leads to glomerulosclerosis by ROS accumulation and triggers interstitial fibrosis via lactic acidosis [128]. Dietary zinc deficiency exacerbates cortical interstitial fibrosis induced by diabetes in rat kidneys [129]. Moreover, zinc deficiency can promote high glucose-induced EMT of normal rat kidney tubular epithelial cells, likely through oxidative stress, phosphoinositide 3-kinase (PI3K)/Akt, p38 MAPK, and ERK activation [130,131,132]. Zinc supplementation mitigates drug-induced cytotoxicity and renal fibrosis, such as that caused by ochratoxin A, in a dose-dependent manner [133].

## 5. Prospects and Challenges

The identification of specific cell death pathways like ferroptosis and cuproptosis has heightened awareness of metal-dependent cell death in diseases, especially renal fibrosis. However, these studies are still in their infancy, with numerous questions and uncertainties remaining to be addressed.

First, it is widely acknowledged that ferroptosis and cuproptosis represent unique cell death forms, distinct from apoptosis, necrosis, and pyroptosis. However, it remains under investigation whether cell death induced by other metal ions, such as zinc, follows similarly specific pathways. Researchers need to further elucidate the specific mechanisms to deepen the comprehension of various metal-dependent cell death mechanisms.

Second, DMT1 is involved in the transport of various metal ions, but its capacity is limited and can become saturated. Competitive inhibition may occur during the transport of different metal ions. If the intake of a particular metal ion exceeds the limitation, the transport of other metals is likely to be compromised. The interactions among different metals are not fully understood; a single metal ion can trigger multiple cell death pathways, while different metal ions may induce the same type of death. Thus, what connections exist among different forms of cell deaths in the presence of various metal ions, and how are signals ultimately mediated in cell deaths? These questions require further investigation. Additionally, many studies focus on changes in the concentration of a single metal ion in specific cell or disease models, often overlooking the potential effects of other metals.

Third, the function of mitochondria in the process of cell death also deserves further analysis. Both ferroptosis and cuproptosis are intimately linked to mitochondrial metabolism, such as the TCA cycle and DHOCH. Zinc serves various functions in mitochondria, such as regulating oxidative phosphorylation, ATP production, and modulating enzyme activity. Further exploration of the impact of metal ions on mitochondrial function and cell metabolism could potentially uncover novel forms of cell death.

Fourth, other cellular organelles, including the lysosomes and ER, are also essential for maintaining metal homeostasis. Interactions within these cellular organelles regulate lysosomal function, ROS production, autophagy, and ion exchange. A deeper understanding of these interactions between cell components will shed further insights into the mechanisms and contexts of metal-dependent cell death.

Fifth, current research on metal-induced cell death and renal fibrosis has predominantly focused on ferroptosis. The roles of other metal-induced cell death pathways in renal fibrosis are still distinguishable. Furthermore, most studies focus on renal tubular epithelial cells in renal fibrosis cell models; research into how other cells, such as renal glomerular mesangial cells and renal glomerular endothelial cells, undergo cell death is lacking. Moreover, designing experimental studies requires a combination of methodologies, including both in vivo and in vitro experiments and analyses of human tissue samples. When selecting experimental methods, molecular techniques such as gene expression analysis, proteomics, and metabolomics may aid in identifying specific cellular pathways and biomarkers associated with metal-dependent cell death. Advanced imaging techniques facilitate cellular visualization, and the ongoing development of emerging technologies like nanomaterials, artificial intelligence, and machine learning continues to deepen our understanding of disease pathogenesis.

Finally, the treatment of renal fibrosis has been a clinical challenge. With the deepening of the research on the mechanism of renal fibrosis, targeting metal-dependent cell death is an effective therapeutic strategy for improving renal fibrosis. It is noteworthy that natural products, including TCM, have garnered significant attention for promising therapeutic drugs for kidney injury induced by metal-dependent cell death due to their diverse biological activities. Particularly, certain TCMs with established anti-inflammatory and antioxidant effects have demonstrated potential in ameliorating renal fibrosis, aligning with the current understanding of their therapeutic mechanisms. Furthermore, exploring the effects of TCM on renal fibrosis holds substantial clinical significance, as it may provide novel therapeutic strategies for managing this condition. With the advancement of technologies like NGS and other advanced technologies, targeted gene therapy may emerge as a potential therapeutic approach for renal fibrosis. NGS allows for the identification of specific genetic mutations and the development of personalized treatment strategies, which can be particularly beneficial in addressing the heterogeneity of renal fibrosis. However, research in this area remains limited, necessitating further exploration to uncover the full potential of targeted gene therapy in treating renal fibrosis. Furthermore, immunotherapy strategies such as T-cell-targeted therapy and B-cell-targeted therapy have shown potential in experimental CKD models. The heterogeneity of macrophages in the kidney and their diverse functions in inflammation and tissue repair suggest that targeting specific macrophage subpopulations could be a therapeutic strategy. The modulation of immune responses through immunotherapy may provide novel therapeutic perspectives and targets for renal fibrosis.

In the future, we anticipate the development and integration of more advanced technologies into clinical practice. The development of targeted therapeutic drugs is also eagerly anticipated, as it could lead to more precise treatments. By elucidating the mechanisms of metal-induced cell death and revealing their role in disease progression, researchers can identify novel intervention targets and formulate targeted treatment strategies to maximize therapeutic efficacy and, to some extent, benefit patients. Of course, such targeted therapies may still bring varying degrees of side effects, but combination or precision therapy may help mitigate adverse reactions. Additionally, achieving precise drug delivery to affected organs remains a significant challenge in the field. Nonetheless, applying these theories in clinical trials and human treatment is still a long-term goal.

## 6. Conclusions

In conclusion, metal-dependent cell death represents a distinct and significant form of PCD with promising applications for various physiological processes and pathological conditions, including renal fibrosis. Further research may yield new insights into the underlying molecular mechanisms of metal-dependent cell death modality and pave the way for novel therapeutic strategies for renal fibrosis.

## Figures and Tables

**Table 1:** The association between metal-dependent cell death and renal fibrosis.

Metal Ion	Cell Death	Drugs and Compounds	Mechanism(s) of Action	Disease	References
Iron	Ferroptosis	Liproxstatin-1	Reduced iron deposition and lipid peroxidation inhibit the downregulation of GPX4 expression	UUO-induced renal fibrosis	[16]
		Astragalus mongholicus bunge and panax notoginseng formula	Inhibiting the long non-coding RNA A33074K22Rik	UUO-induced renal fibrosis	[96]
		Kaempferitrin	Inhibiting NOX4-mediated tubular ferroptosis	UUO-induced renal fibrosis	[97]
		Nobiletin	Inhibiting ferroptosis	UUO-induced renal fibrosis	[98]
		Tectorigenin	Inhibiting Smad3-mediated ferroptosis	UUO-induced renal fibrosis	[99]
		Baicalein	Inhibiting ferroptosis	UUO-induced renal fibrosis	[100]
		Tocilizumab mimotope	Inhibiting ferroptosis	UUO-induced renal fibrosis	[101]
		Formononetin	Suppress Smad3/ATF3/SLC7A11 signaling	UUO and FA-induced renal fibrosis	[102]
		FG-4592	Decreasing ferroptosis via Akt/GSK-3*β*/Nrf2	FA-induced kidney injury	[103]
		Ferrostatin-1	Inhibit HIF-1α/HO-1 pathway	DN	[104]
		Hederagenin	Regulating Smad3/NOX4/SLC7A11 signaling	DN	[105]
		Ginkgolide B	Inhibiting GPX4 ubiquitination	DN	[106]
		Rhein	Regulating the Rac1/NOX1/β-Catenin Axis	DN	[107]
		Shenkang Pills	Subduing the HIF-1α/HO-1 signaling pathway	DN	[108]
		Atorvastatin	Inhibiting oxidative stress and ferroptosis	DN	[109]
		tRNA-derived fragments 3	Targeting zinc finger protein 281	DN	[110]
		Endoplasmic reticulum stress	Triggered the XBP1-Hrd1-Nrf2 pathway	DN	[111]
		Homeobox D10	Inhibiting NOX4 transcription	CKD	[112]
		Vitexin	Activate Nrf2/HO-1 pathway	CKD	[113]
		Indoxyl Sulfate	Triggers intracellular iron accumulation and ROS generation	CKD	[114]
		Fisetin	Inhibiting ACSL4-mediated tubular ferroptosis	CKD	[115]
		Cisplatin and deferoxamine mesylate	Inducing or inhibiting ferroptosis	5/6 nephrectomy induced-CKD	[13]
		Sirtuin7	Promoting KLF15/Nrf2 signaling	Hypertensive nephropathy	[116]
		Stimulator of interferon genes	Inducing ACSL4-dependent ferroptosis	Hypertensive nephropathy	[117]
		Ferrostatin-1	Suppress ferroptosis	Hyperuricemic nephropathy	[118]
		Ferrostatin-1	Suppress ferroptosis	Oxalate-induced renal fibrosis	[17]
		TNF-α	Inducing IRF1/ZNF350/GPX4 signaling	Renal interstitial fibrosis	[119]
		Melatonin and Zileuton	Inhibition AKT/mTOR/NRF2 Signaling	Renal fibrosis	[120]
		Aging macrophage	Regulating ferroptosis	Renal fibrosis	[121]
		Low-intensity pulsed ultrasound	Inhibiting Nrf2/keap1/HO-1 signaling	Renal fibrosis	[122]
		Puerarin	Inhibiting TLR4/NOX4 pathway	Ischemia/reperfusion injury	[123]
		High-fat diet	Inducing ferroptosis	Obesity-induced renal injury	[124]
Copper		Intercellular extracted copper	Activated lysyl oxidase to enhance the crosslinking of collagen and elastin	Renal fibrosis	[22]
		Mitochondrial copper overload	Inhibit pyruvate dehydrogenase activity	Renal fibrosis	[125]
		COX17	Maintain mitochondrial copper homeostasis	Renal fibrosis	[126]
Zinc		Zinc deficiency	Increasing inflammation	Renal fibrosis	[127]
		Zinc deficiency	Podocyte damage via accumulation of ROS	Renal interstitial fibrosis	[128]
		Dietary zinc	Modifies renal pathology	DN	[129]
		Zinc deficiency	Oxidative stress, PI3K/Akt, p38 MAPK and ERK activation	DN	[130,131,132]
		Ochratoxin A	Zinc application attenuated cell toxicity	Renal fibrosis	[133]

## Data Availability

Not applicable.

## Abbreviations

acyl-CoA synthetase long-chain	ACSL
buthionine sulfoximine	BSO
copper affinity transporter 1	CTR1
cytochrome C oxidase	COX
coenzyme Q_10_	CoQ_10_
chronic kidney disease	CKD
divalent metal-ion transporter 1	DMT1
dihydrolipoamide S-acetyltransferase	DLAT
dihydroorotate dehydrogenase	DHODH
diabetic nephropathy	DN
extracellular matrix	ECM
extracellular regulated protein kinases	ERK
electron transport chain	ETC
epithelial-mesenchymal transition	EMT
ferric iron	Fe^3+^
ferrous iron	Fe^2+^
ferredoxin 1	FDX1
glutathione peroxidase 4	GPX4
glutathione	GSH
hemeoxygenase-1	HO-1
Interleukin-6	IL-6
labile iron pool	LIP
lipoic acid synthetase	LIAS
nuclear factor erythroid 2-related factor 2	Nrf2
NADPH oxidase 4	NOX4
iron-sulfur cluster protein	Fe-S cluster protein
iron regulatory protein	IRP
ischemia-reperfusion	IR
programmed cell death	PCD
polyunsaturated fatty acid	PUFA
protein kinase B	AKT
reactive oxygen species	ROS
solute carrier family	SLC
superoxide dismutase 1	SOD1
tricarboxylic acid	TCA
traditional Chinese medicine	TCM
unilateral ureteral obstruction	UUO
